# Genome sequence and assembly of endophyte *Pseudomonas lini* Can06R isolated from the roots of *Chenopodium album* L.

**DOI:** 10.1128/mra.00834-26

**Published:** 2026-09-28

**Authors:** Vladimir K. Chebotar, Maria S. Gancheva, Margarita V. Khudyaeva, Oksana V. Keleinikova, Maria E. Baganova, Alexander N. Zaplatkin, Veronika N. Pishchik, Elena P. Chizhevskaya

**Affiliations:** 1 All-Russia Research Institute for Agricultural Microbiology, St. Petersburg, Russia; 2 Saint Petersburg State University, St. Petersburg, Russia; University of Strathclyde, Glasgow, United Kingdom

**Keywords:** genome sequence, endophyte, *Pseudomonas lini*, roots, *Chenopodium album*

## Abstract

The genome of endophyte *Pseudomonas lini* strain Can06R isolated from surface-sterilized roots of *Chenopodium album* L. was sequenced using an Illumina NextSeq 1000 platform and assembled with SPAdes. The assembly is 6,302,676 bp in length, has a GC content of 58.8%, and contains 5,730 predicted genes.

## ANNOUNCEMENT

The roots of drought-tolerant *Chenopodium album* L. (Stavropol region, Russia; 44°33′43″ N, 44°49′59″ E) were used to isolate endophytic bacteria with potential plant growth-promoting traits. A composite 5-g root sample from three plants was cleaned, surface-sterilized by treating sequentially with tap water (30 s), 70% ethanol (5 min), 15% H₂O₂ (10 min), and sterile water (5 × 2 min), and crushed in 2.5 mL of sterile 0.85% NaCl. The resulting homogenate (0.5 mL) was plated on R2A medium (Thermo Fisher Scientific, USA) and incubated at 28°C for 72 h. The obtained strain, designated Can06R, was purified by repeated streaking on R2A under the same incubation conditions. Strain Can06R was grown in R2A medium at 30°C overnight for DNA extraction. DNA was extracted from a single colony using the Wizard Genomic DNA Purification Kit (Promega, USA). Libraries were constructed with 100 ng of DNA using the KAPA HyperPlus Kit (Roche, Switzerland), assessed with the High-Sensitivity DNA Kit (Agilent Technologies, USA), and sequenced on an Illumina NextSeq 1000 platform (2 × 150 bp) using P1 reagents (300 cycles) and a 2% PhiX control, yielding 1,907,717 read pairs.

After quality filtering with fastp v0.23.4 (1), reads were assembled with SPAdes v3.14.1 (2) (--careful). The resulting assembly was scaffolded against the *Pseudomonas lini* DSM 16768 reference genome (GCF_900104735.1)—the closest relative among all *Pseudomonas* RefSeq genomes (3) (accessed May 2026) according to FastANI v1.34 (4) (95% average nucleotide identity [ANI])—using Ragtag v2.1.0 (5), and scaffolds shorter than 500 bp were removed. Coverage was determined with samtools coverage v1.19.2 (6) after read mapping with BWA v0.7.17 (7). Assembly quality metrics were assessed by QUAST v5.3.0 (8), CheckM v1.2.4 (*Pseudomonas* marker set) (9), and BUSCO v5.5.0 (gammaproteobacteria_odb10) (10). The final assembly consists of five scaffolds from 52 contigs with a total length of 6,302,676 bp, GC content of 58.8%, an N50 of 6,294,677 bp, and an average coverage of 89.4×. BUSCO completeness was 100.0% (C: 100.0% [S: 99.2%, D: 0.8%], F: 0.0%, M: 0.0%, *n*: 366), while CheckM completeness was 99.68% (contamination: 0.48%). Annotation with PGAP v6.11 (11) yielded 5,730 total genes (5,570 protein-coding). Unless otherwise specified, default software parameters were applied.

*P. lini* strain GHM32 was recently shown in field trials to act as a diazotroph, improving nitrogen use efficiency and yield in rice (12); therefore, strains of this species are of considerable interest, particularly those isolated from drought-tolerant plants. Sequencing the genome of our strain obtained from the roots of the drought-tolerant plant *C. album* will facilitate comparative identification of genetic traits potentially contributing to nitrogen supply and stress mitigation under water-limited conditions.

## Data Availability

The genome sequence and raw reads were deposited in NCBI and can be accessed under BioProject PRJNA1470951 (SRA SRR38851304, assembly GCA_057837355.1).

## References

[1] Chen S. 2023. Ultrafast one-pass FASTQ data preprocessing, quality control, and deduplication using fastp. Imeta 2:e107. doi:10.1002/imt2.107 PMC10989850 38868435

[2] Prjibelski A, Antipov D, Meleshko D, Lapidus A, Korobeynikov A. 2020. Using SPAdes de novo assembler. Curr Protoc Bioinformatics 70:e102. doi:10.1002/cpbi.102 32559359

[3] O’Leary NA, Wright MW, Brister JR, Ciufo S, Haddad D, McVeigh R, Rajput B, Robbertse B, Smith-White B, Ako-Adjei D, et al.. 2016. Reference sequence (RefSeq) database at NCBI: current status, taxonomic expansion, and functional annotation. Nucleic Acids Res 44:D733–D745. doi:10.1093/nar/gkv1189 PMC4702849 26553804

[4] Jain C, Rodriguez-R LM, Phillippy AM, Konstantinidis KT, Aluru S. 2018. High throughput ANI analysis of 90K prokaryotic genomes reveals clear species boundaries. Nat Commun 9:5114. doi:10.1038/s41467-018-07641-9 PMC6269478 30504855

[5] Alonge M, Soyk S, Ramakrishnan S, Wang X, Goodwin S, Sedlazeck FJ, Lippman ZB, Schatz MC. 2019. RaGOO: fast and accurate reference-guided scaffolding of draft genomes. Genome Biol 20:224. doi:10.1186/s13059-019-1829-6 PMC6816165 31661016

[6] Danecek P, Bonfield JK, Liddle J, Marshall J, Ohan V, Pollard MO, Whitwham A, Keane T, McCarthy SA, Davies RM, Li H. 2021. Twelve years of SAMtools and BCFtools. Gigascience 10:giab008. doi:10.1093/gigascience/giab008 PMC7931819 33590861

[7] Li H. 2013. Aligning sequence reads, clone sequences and assembly contigs with BWA-MEM. arXiv. doi:10.48550/arXiv.1303.3997

[8] Gurevich A, Saveliev V, Vyahhi N, Tesler G. 2013. QUAST: quality assessment tool for genome assemblies. Bioinformatics 29:1072–1075. doi:10.1093/bioinformatics/btt086 PMC3624806 23422339

[9] Parks DH, Imelfort M, Skennerton CT, Hugenholtz P, Tyson GW. 2015. CheckM: assessing the quality of microbial genomes recovered from isolates, single cells, and metagenomes. Genome Res 25:1043–1055. doi:10.1101/gr.186072.114 PMC4484387 25977477

[10] Manni M, Berkeley MR, Seppey M, Simão FA, Zdobnov EM. 2021. BUSCO update: novel and streamlined workflows along with broader and deeper phylogenetic coverage for scoring of eukaryotic, prokaryotic, and viral genomes. Mol Biol Evol 38:4647–4654. doi:10.1093/molbev/msab199 PMC8476166 34320186

[11] Tatusova T, DiCuccio M, Badretdin A, Chetvernin V, Nawrocki EP, Zaslavsky L, Lomsadze A, Pruitt KD, Borodovsky M, Ostell J. 2016. NCBI prokaryotic genome annotation pipeline. Nucleic Acids Res 44:6614–6624. doi:10.1093/nar/gkw569 PMC5001611 27342282

[12] Quadriya H, Rajendran G, Mir MI, Surekha K, Hameeda B. 2024. Role of *Pseudomonas lini* and *Brevundimonas nasdae* to enhance nitrogen use efficiency (NUE) and yield of *Oryza sativa* L. Int J Plant Prod 18:271–287. doi:10.1007/s42106-024-00289-0

